# Immersive virtual reality as support for the mental health of elderly women: a randomized controlled trial

**DOI:** 10.1007/s10055-023-00797-w

**Published:** 2023-05-07

**Authors:** Błażej Cieślik, Karolina Juszko, Pawel Kiper, Joanna Szczepańska-Gieracha

**Affiliations:** 1 Healthcare Innovation Technology Lab, IRCCS San Camillo Hospital, Venice, Lido, Italy; 2 Faculty of Physiotherapy, Wroclaw University of Health and Sport Sciences, Wrocław, Poland

**Keywords:** Virtual therapeutic garden, Hypnosis, VRET, Mood disorders, Head-mounted display, Exposure therapy

## Abstract

Several forms of virtual reality (VR) have shown promise in treating mental disorders. However, there is a lack of research investigating the use of multicomponent immersive VR. Therefore, this study aimed to evaluate the effectiveness of an immersive virtual reality (IVR) intervention that incorporated Japanese garden aesthetics, relaxation, and elements of Erickson’s psychotherapy in alleviating depression and anxiety symptoms among elderly women. Sixty women with depressive symptoms were randomly assigned to one of two treatment groups. Both groups received eight (twice a week for four weeks) low-intensity general fitness training sessions. The IVR group (*n* = 30) received eight additional VR-based relaxation sessions, whereas the control group (*n* = 30) received eight group relaxation. As outcome measures, the geriatric depression scale (GDS; primary) and Hospital Anxiety and Depression Scale (HADS; secondary) were administered before and after the interventions. The protocol was registered in the ClinicalTrials.gov PRS database (Registration number: NCT05285501). Patients receiving IVR therapy exhibited a greater significant reduction in the GDS (adjusted mean post-difference of 4.10; 95% CI = 2.27–5.93) and HADS (2.95; 95% CI = 0.98–4.92) scores than those receiving the control intervention. In conclusion, IVR with elements of psychotherapy, relaxation, and garden aesthetics may alleviate the severity of depression and anxiety symptoms in elderly women.

## Introduction

In the general population, one in five people experience one episode of depression at some point in their lifetime (Malhi and Mann 2018). Estimates of the prevalence of depression in adults are inconsistent and depend, among others, on etiology, place of residence, severity, gender or age (Luppa et al. 2012). Despite the wide variation in estimates, it is evident that late-life depression is common, with the prevalence of depressive disorders ranging from 4 to 37% (Luppa et al. 2012). Importantly, women are twice as likely as men to suffer from an episode of depression (Sassarini 2016) and the female preponderance in depression continues into old age (Kuehner 2017). Potential explanations for the gender gap in depressive symptoms are still scarce; nevertheless, the causes are sought in genetic factors, childhood gender-based violence or altered hormonal responses (Kuehner 2017).

The search for non-pharmacological forms of interventions for depression support in older adults has been studied for several years. In most studies, older adults who received various relaxation interventions experienced greater improvements in depression and anxiety than controls (Klainin-Yobas et al. 2015). For instance, progressive muscle relaxation exercises were effective in reducing depression levels (Gökşin and Aşiret 2021). Similar results were obtained with a mindfulness-based stress reduction intervention compared to a waiting-list control immediately after intervention (Li and Bressington 2019). Other studies concluded that music therapy added to treatment as usual (TAU) appears to improve depressive and anxiety symptoms compared to TAU alone (Aalbers et al. 2017; Tang et al. 2020).

One of the environmental factors that seem to affect the well-being and longevity of older people is neighborhood greenness and green spaces (Ji et al. 2019; Perrino et al. 2019). A recent study conducted in 881 Japanese neighborhood districts (126,878 participants) concluded that lower odds of depression were associated with a large amount of green space among older adults (Nishigaki et al. 2020). Banay et al. (2019) analyzed 38,947 women and showed that living in the highest quintile of residential greenness was associated with a 13% reduction in the risk of depression compared with the lowest quintile. For this reason, an ever-growing number of studies using various forms of green areas to improve mood disorders are justified. A recent systematic review conducted by Taylor et al. concluded that nature-based interventions have positive psychological effects on people with long-term conditions (Taylor et al. 2022). Furthermore, various horticultural therapies have shown promising effects on physical functioning and the psychological health of older adults (Lin et al. 2022). Although gardening requires active participation in the care of plants, watching them can also have a relaxing effect (Goto et al. 2013). A previous study showed that sitting in front of a garden could affect both mood and cardiac physiology in elderly individuals (Goto et al. 2013).

However, access to green spaces or gardens is not always possible. Certain hospital inpatients and individuals with disabilities or immobility, for example, have limited access to greenness (Landeiro et al. 2016). In addition, the COVID-19 pandemic and forced lockdowns have shown that under certain conditions the possibility of locomotion and going outside may be restricted (Gloster et al. 2020). In such cases, virtual reality (VR) could be a useful solution, as it allows isolated individuals to engage in new experiences and explore environments to which they might not otherwise have access (Slater 2018). Based on the degree of user engagement, VR is typically divided into immersive, which replaces the user’s physical reality with head-mounted displays (HMD) or cave automatic virtual environments (CAVE), and non-immersive, which adds virtual elements to the user's physical reality through screen displays (Domínguez-Téllez et al. 2020). An increasing number of studies have focused on exploring virtual environments and VR to support mental health in various psychiatric diseases (Cieślik et al. 2020). For instance, for anxiety and phobias, the commonly used therapy is Virtual Reality Exposure Therapy (VRET), which involves exposing patients to simulated situations that replicate the triggers of their fears and anxieties in real life (Carl et al. 2019). In pain management, VR works by distracting patients from physical discomfort, inducing relaxation, and altering their perception of pain (Goudman et al. 2022). VR therapy for depression involves immersing patients in virtual environments, such as tranquil natural landscapes, guided meditations, and social scenarios, that are designed to promote positive emotions and improve social skills while reducing symptoms of depression (Freeman et al. 2017). Ten years ago, Baños et al. proposed two virtual environments that simulated natural environments for psychological support (Baños et al. 2013). In 2020 a study protocol appeared, which adapted the idea of a therapeutic garden to immersive VR in order to improve well-being during the COVID-19 pandemic (Riva et al. 2020). A recent mini-review on access to nature through VR concluded that the application of virtual nature, simulated green exercises and diverse virtual natural environments could induce a relaxation effect and may be a topic for further study (Li et al. 2021).

VR is a versatile medium, and its effectiveness depends largely on the software used. Immersive head-mounted display (HMD) VR can be particularly effective in creating green environments and incorporating elements of stress-reduction activities and music. Therefore, this study aimed to evaluate the effectiveness of Immersive Virtual Reality (IVR) that incorporates Japanese garden aesthetics and elements of Erickson’s psychotherapy in alleviating depression and anxiety symptoms in elderly women who participated in support groups. We hypothesized that IVR would be more effective than group relaxation in reducing anxiety and depressive symptoms in elderly women.

## Materials and methods

### Participants and study setting

**Fig. 1:**
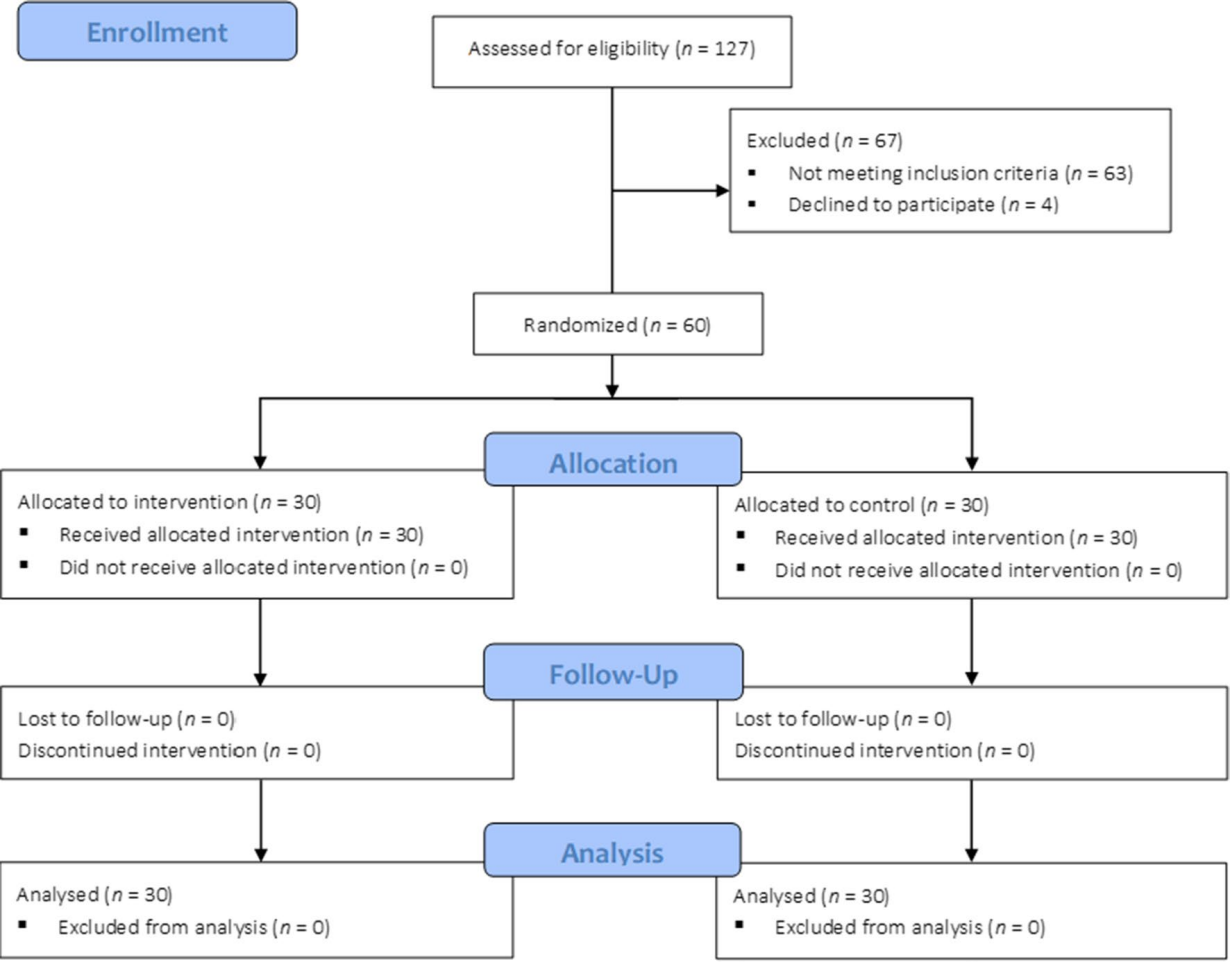
Study flow diagram

The study was conducted at the Foundation for Senior Citizen Activation SIWY DYM, in Wroclaw, Poland. The foundation promotes a healthy lifestyle and offers the organization of physical exercises and psychological support for elderly women. After an initial eligibility evaluation, 60 women with depressive symptoms were randomly assigned to one of two treatment groups (Fig. 1). The inclusion criteria were as follows: age 60–85 years and a 30-item Geriatric Depression Scale (GDS-30) score of < 10 or a Hospital Anxiety and Depression Scale (HADS) score of < 8. We excluded participants with: a score lower than 24 points on the Mini Mental State Examination (MMSE); aphasia; severe loss of sight or hearing that made it impossible to assess cognitive function; participation in other therapeutic projects or individual psychotherapy; and pharmacological depression treatment.

This study was designed as a parallel-group randomized controlled trial with a masked outcome assessor and measures repeated at two time points: pre- and post-intervention. Participants were randomly assigned (1:1) to two groups using the block randomization method: 30 individuals were included in the experimental group (IVR group) and 30 served as the active comparator control group. The randomization sequence was created using a computer software program and patients were enrolled using sealed sequentially numbered envelopes. The principal investigator concealed the allocation sequence. To ensure blinding of the treatment allocation and randomization procedures, the researcher responsible for randomization was independent of the assessors.

The design followed the recommendations for the third phase (VR3) of health clinical trials using VR, focusing on the effectiveness of the proposed treatment modality with respect to the control group (Birckhead et al. 2019). The study protocol was reviewed and approved (Ref. No. 32/2019) by the Institutional Review Board of Wroclaw University of Health and Sport Sciences (Wroclaw, Poland) and was registered in the ClinicalTrials.gov database before participant recruitment began (Registration number: NCT05285501). All participants provided their written informed consent to participate in this study.

### Outcomes

The 30-item Geriatric Depression Scale (GDS-30) was used as the primary outcome measure. This is a self-rated screening tool that is recommended as a depression screening tool for elderly individuals (Lopez et al. 2010). A score of 11 or greater indicates an increasing severity of depression. The scale has high reliability (Cronbach’s *α* = 0.69–0.99) and validity (Mitchell et al. 2010).

The Hospital Anxiety and Depression Scale Anxiety (HADS) was used as a secondary outcome measure. The HADS is a 14-item self-report questionnaire designed to screen for anxiety (HADS-A subscale; seven items) and depression (HADS-D subscale; seven items) in patients in non-psychiatric settings, with a cut-off point of 8/21 for both subscales. Cronbach’s α ranges from 0.78 to 0.93, and the test–retest correlation was *r* = 0.80 (Bjelland et al. 2002).

### Interventions

Over four consecutive weeks, twice a week, both groups participated in general fitness training (GFT), totaling eight sessions overall. Additionally, the IVR group received eight sessions of immersive virtual therapeutic garden (IVTG) intervention, whereas the control group received eight sessions of relaxation and psychoeducation.

Each GFT session was conducted in a gym and lasted 40 min, with groups of approximately 10–12 women. A single GFT session comprised low-intensity general fitness exercises, with most of the exercises performed in sitting and standing positions. The GFT sessions were performed by a physiotherapist and consisted of aerobic (general warm-up), musculoarticular (strengthening muscles and joint range of motion) and stabilizing exercises (improved spatiovisual coordination).

**Fig. 2:**
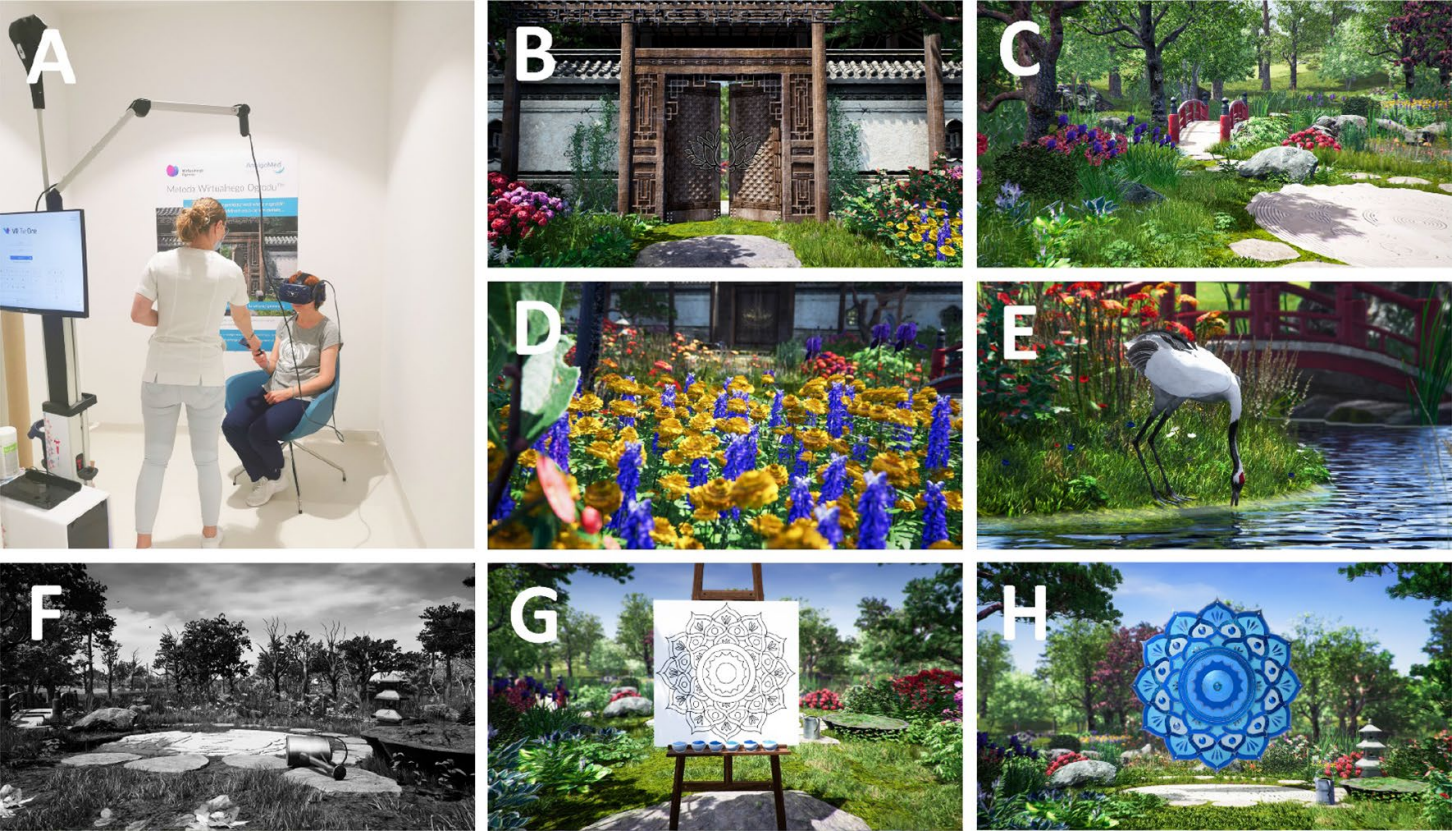
IVTG screen captures: **a** patient setting; **b** gate leading to the garden; **c**, **d**, **e** garden decor elements; **f** initial stage of therapy; **g** mandala coloring task; **h** final colored mandala

The IVR group received eight sessions (twice a week, 20 min each) of IVTG with the VRTierOne device (Stolgraf^®^, Stanowice, Poland). The hardware consisted of VR HTC VIVE goggles (2017) and two HTC VIVE controllers (Fig. 2a). The main purpose of IVTG was, due to immersive VR, to transfer attention to a peaceful (virtual) environment, experience a state of relaxation, and help patients recognize their psychological resources. At the beginning of the session, the patient was placed in front of a garden door (Fig. 2b). After a few minutes, the door to the garden opened, and the patient moved inside and was encouraged to observe the elements of the garden (Fig. 2c, d, e). The garden, initially neglected (Fig. 2f), grew livelier and more colorful with each session. In the middle of the session, a black and white mandala appeared in front of the participant (Fig. 2g, h). The patient’s task was to color it using the controllers. The therapeutic effect of the IVTG is based on four elements: aspects of Erickson’s psychotherapy, relaxing music, cognitive stimulation and a green garden environment based on Japanese esthetics. During therapy, the reader instructs the patients on how to relax and increase their self-awareness using posthypnotic suggestions. This procedure is based on the assumptions of Erickson’s psychotherapy, relies primarily on metaphorical communication and uses specific symbolisms (Matthews 2000). The music used was composed by the collaboration of a music therapist and a music composer. It is relaxing and becomes more joyful as the therapy progresses. Cognitive stimulation includes the task of coloring mandalas using controllers and is designed to involve the patient by active participation in therapy. The final element is the garden itself; staying in a green and vibrant environment is meant to evoke positive associations and influence the mood of the participant. A detailed description of the assumptions of the VRTierOne device was included in our previous study (Szczepańska-Gieracha et al. 2021a).

The control group received eight sessions (two times a week, 20 min each) of group relaxation (10 min) and psychoeducation (10 min). Relaxation consisted of breathing exercises with muscle relaxation and guided imaginary, which were aimed at promoting a positive vision of oneself. Psychoeducation comprised mini-lectures on mental well-being and psychohygiene.

### Data analysis

The sample size for this study was calculated using G*Power 3.1.9.4 software (Heinrich-Heine-University Düsseldorf, Germany) with a priori power analysis for covariance analysis (ANCOVA) (Faul et al. 2007). Based on the results of the primary outcome (GDS) of our previous pilot study, we assumed an effect size of 0.62 (pilot partial eta squared = 0.28)(Szczepańska-Gieracha et al. 2021a). With a minimum significance (*α*) of 0.05 and statistical power (1 − *β*) of 0.99, 50 participants were needed to reach statistical significance. Furthermore, we assumed a 20% dropout rate; thus, a total of 60 participants were included.

Data were analyzed using JASP version 0.16.3 (University of Amsterdam, The Netherlands). Descriptive statistics for categorical variables were reported as frequency counts and percentages. For continuous variables, we report the mean and standard deviation (SD). The Shapiro–Wilk test showed a normal distribution of quantitative data. Baseline demographic variables were compared between groups using unpaired *t*-tests (for continuous variables) and *χ*^2^ tests (for categorical data). A paired *t-*test was used to analyze the effects of the intervention (pre- vs. post-intervention). To verify the magnitude of the differences, Cohen’s *d* was calculated, where 0.20–0.49 was considered small, 0.50–0.79 as moderate and ≥ 0.80 as large (Cohen 1992). ANCOVA was used to compare the groups before and after the intervention, with baseline values as covariates. For ANCOVA, partial eta squared (*η*p^2^) was calculated as the effect size, with a value of < 0.5 considered a small effect, 0.6–0.13 as a medium effect and ≥ 0.13 as a large effect. Statistical significance was established at *α* < 0.05.

## Results

Of the 127 available potential participants, 60 were randomized for the study based on the inclusion criteria. All enrolled participants completed the study (Fig. 1). As presented in Table 1, no statistically significant differences were observed between the IVR and control groups at baseline.

**Table 1:** Participants baseline characteristics

Variable	Overall	IVR	Control	*P* value
*n*	60	30	30	–
Age, years	68.15 (5.53)	68.77 (5.57)	67.53 (5.51)	0.39^a^
Body mass, kg	76.70 (14.77)	75.78 (12.05)	77.63 (17.22)	0.63^a^
Body height, m	1.64 (0.08)	1.64 (0.08)	1.65 (0.09)	0.53^a^
Body mass index, kg/m^2^	28.44 (5.01)	28.40 (4.52)	28.48 (5.54)	0.95^a^
Normal (BMI 18.5–24.9), *n* (%)	14 (23.33)	6 (20.00)	8 (26.67)	0.54^b^
Overweight (BMI 25–29.9), *n* (%)	27 (45.00)	14 (46.67)	13 (43.33)	0.79^b^
Obese (BMI > 30), *n* (%)	19 (31.67)	10 (33.33)	9 (30.00)	0.78^b^
*Marital status, n (%)*				
Married	32 (53.33)	15 (50.00)	17 (56.67)	0.60^b^
Single	5 (8.33)	3 (10.00)	2 (6.67)	0.63^b^
Widowed	23 (38.33)	12 (40.00)	11 (36.67)	0.79^b^
*Education, n (%)*				
Primary/vocational	27 (45.00)	14 (46.67)	13 (43.33)	0.79^b^
Secondary	21 (35.00)	9 (30.00)	12 (40.00)	0.41^b^
Higher	12 (20.00)	7 (23.33)	5 (16.67)	0.51^b^

*IVR* immersive virtual reality, *BMI* body mass index

^a^*t-*test

^b^Chi-square test

Statistically significant differences were observed between the groups in the post-treatment assessment (Table 2); however, these differences were significantly higher in the IVR group. For the primary outcome measure, the GDS score was reduced by 44.05% in the IVR group whereas in the control group it was reduced by 13.81%. However, the between-group adjusted mean difference for post-treatment evaluation was 4.10 (95% CI = 2.27–5.93; *P* < 0.001). A similar pattern was observed for the secondary outcome measure. In the IVR group, the total HADS score decreased significantly (26.95%), along with the HADS-A (31.98%) and HADS-D (by 21.09%). In the control group, a significant reduction in the total HADS (10.20%) and HADS-A (14.40%) was observed; the HADS-D did not change significantly in the post-treatment evaluation. For the secondary outcome measure, the adjusted mean difference in between-group comparison demonstrated significant differences: 2.95 (95% CI 0.98, 4.92), 1.68 (95% CI 0.37, 3.00) and 1.22 (95% CI 0.00, 2.44) for HADS, HADS-A and HADS-D, respectively.

**Table 2:** Mean values (SD) of primary and secondary outcomes

Outcome	IVR (*n* = 30)				Control (*n* = 30)				Between-group comparison	
	Baseline	Post-treatment	Cohen’s* d* (95% CI)	*P* value	Baseline	Post-treatment	Cohen’s* d* (95% CI)	*P* value	Adjusted mean difference (95% CI)	*P* value
GDS	13.10 (4.26)	7.33 (3.88)	1.86 (1.26–2.45)	< 0.001	13.27 (3.80)	11.57 (5.49)	0.42 (0.04–0.79)	0.029	4.10 (2.27–5.93)	< 0.001
HADS	15.10 (5.07)	11.03 (3.98)	0.93 (0.50–1.36)	< 0.001	16.37 (4.51)	14.70 (5.18)	0.40 (0.02–0.76)	0.039	2.95 (0.98–4.92)	0.004
HADS-A	8.13 (3.66)	5.53 (2.94)	0.91 (0.48–1.33)	< 0.001	9.03 (3.07)	7.73 (3.35)	0.45 (0.07–0.82)	0.020	1.68 (0.37–3.00)	0.013
HADS-D	6.97 (2.59)	5.50 (2.52)	0.61 (0.21–0.99)	0.002	7.37 (2.44)	6.97 (3.06)	0.15 (0.21–0.51)	0.412	1.22 (0.00–2.44)	0.049

*GDS* geriatric depression scale, *HADS* hospital anxiety and depression scale, *HADS-A* anxiety subscale of the HADS, *HADS-D* depression subscale of the HADS, *SD* standard deviation, *IVR* immersive virtual reality

When comparing data between groups with baseline values as a covariable, significant group × time interactions were revealed for the GDS (*F* = 20.08, *P* < 0.001, *η*p^2^ = 0.26), HADS (*F* = 9.02, *P* = 0.004, *η*p^2^ = 0.14), HADS-A (*F* = 6.60, *P* = 0.013, *η*p^2^ = 0.10) and HADS-D (*F* = 4.01, *P* = 0.049, *η*p^2^ = 0.06) scores (Table 3).

**Table 3:** ANCOVA results

Outcome	Mean square	*F*	*P* value	*η*p^2^
*GDS*				
Group	252.15	20.08	< 0.001	0.26
Baseline GDS	596.25	47.48	< 0.001	0.45
*HADS*				
Group	128.35	9.02	0.004	0.14
Baseline HADS	425.87	29.92	< 0.001	0.34
*HADS-A*				
Group	41.80	6.60	0.013	0.10
Baseline HADS-A	216.50	34.20	< 0.001	0.37
*HADS-D*				
Group	22.22	4.01	0.049	0.06
Baseline HADS-D	138.65	25.02	< 0.001	0.30

*GDS* geriatric depression scale, *HADS* hospital anxiety and depression scale, *HADS-A* anxiety subscale of the HADS, *HADS-D* depression subscale of the HADS

## Discussion

In accordance with the purpose of this study, we confirmed our hypothesis that an IVR is more effective than group relaxation for reducing anxiety and depressive symptoms among elderly women participating in support groups. The adjusted mean difference for depression (GDS) was 4.10 points, with a large effect size (*η*p^2^) of 0.26. This finding was supported by the results for the secondary outcome measure (HADS-D) but with a smaller effect size (*η*p^2^ = 0.06). Importantly, due to the fact that these two outcomes differ in sensitivity and question focus, this confirms the reliability of the achieved effectiveness (Campbell et al. 2015). Moreover, both outcomes improved in the control group, confirming the effectiveness of the control intervention. This is important because as an active comparator we used a previously proven multimodal therapeutic intervention, which itself reduces the symptoms of depression and stress (Szczepańska-Gieracha et al. 2019; Morga et al. 2021). Despite this, the IVR intervention was significantly more effective at alleviating symptoms of depression and anxiety.

The source of effectiveness of the IVR should be determined based on its assumptions. The therapeutic effect of a IVTG is based mainly on aspects of Erickson's psychotherapy, but also on relaxing music, cognitive stimulation and a green garden environment. Each of these stimuli has proven to be effective in supporting mental health (Aalbers et al. 2017; Banay et al. 2019). However, in our opinion, the most important aspect is the implementation of aspects of Ericksonian psychotherapy. The therapeutic foundation of this approach is based on indirect and metaphoric communication that reflects the essence of the patient’s problem (Lynn et al. 1993). Ericksonian psychotherapy uses hypnotic suggestions, positive reinforcement and metaphoric storytelling to guide patients in finding solutions to their problems (Larkin 2014; Moss 2019). Furthermore, the garden esthetics used in this study were based on the concept of Japanese gardens. The spatial arrangement of a garden requires close inspection of the area (Goto et al. 2020). According to the authors, the relaxing mechanisms of Japanese gardens lie in evoking memories of viewing certain landscape images, thereby inducing a sense of comfort and decreasing the heart rate (Goto et al. 2020).

The results obtained in this study are consistent with our previous research on IVTG therapy in other rehabilitation settings, indicating that the intervention had a positive impact on mental health outcomes. Specifically, the applied intervention significantly reduced the symptoms of depression by 46%, 21%, and 24% for stroke, cardiac, and pulmonary patients, respectively, suggesting that the intervention could be effective in patients with different medical conditions (Rutkowski et al. 2021; Jóźwik et al. 2021; Szczepańska-Gieracha et al. 2021b; Kiper et al. 2022). Other studies using VR greenery have shown similar effectiveness (Boffi et al. 2022): Chan et al. (2021) showed that a short walk in a virtual forest improved the emotional state of the elderly; a recent mixed-method feasibility study demonstrated the usefulness of different nature-based VR environments in mood improvement (Kalantari et al. 2022); and the use of the VR garden idea had positive effects on affect, well-being and stress in the face of isolation related to the COVID-19 pandemic (Malighetti et al. 2022; Meyer et al. 2022; Pallavicini et al. 2022). Nevertheless, the main difference between the above research and this study was the additional implementation of psychotherapeutic elements in our intervention.

### Bigger picture

In the field of cyberpsychology, exactly 10 years ago Riva et al. (2012) proposed a combination of new technologies and positive psychology. The authors wondered how digital technologies could help to shape positive human functioning. Consequently, research on the use of VR as a medium for psychotherapy has emerged (Botella et al. 2012). The most commonly used forms of therapy transferred to VR are cognitive behavioral therapy and VR exposure therapy (VRET) (Wu et al. 2021; Baghaei et al. 2021). However, to our knowledge, our study is the first attempt to transfer Ericksonian psychotherapy elements to an immersive VR environment. Freeman et al. asked: ‘Can key theory-driven psychological treatment techniques (beyond simple exposure) be successfully delivered in VR?’ (Freeman et al. 2017). We hope that this study provides testimony to the possibility of using VR for different forms of psychotherapy, such as the Ericksonian approach or other indirect psychological treatments.

In addition, this study has clinical utility. Similar solutions can be used under all conditions in which access to real green areas and psychological support is limited. In hospital wards, the traditional forms of relaxation played by compact disks might be replaced by VR-based interventions. Patients who experience long-term immobilization could also benefit from immersion in an IVTG. Finally, it holds great promise as a non-pharmacological therapy for improving mental health and well-being in everyday home settings.

### Study limitation

The greatest limitation of this study was the use of questionnaires as outcome measures. Insight into more objective measures would help to understand the mechanisms and nature of induced relaxation. Therefore, further studies should focus on heart rate variability, electroencephalography or cytokines (especially interleukin-1), as these are commonly useful for measuring and capturing relaxation effects. Another limitation of this study is related to the above discussion. In our study, we included individuals based on the results of the depression and anxiety questionnaire without a psychiatric diagnosis. Evaluation of the effectiveness of this solution would be interesting for patients with major depressive disorder. Moreover, when assessing psychological symptoms, it is worth examining whether the intervention has a long-term impact; therefore, the lack of follow-up assessment in this study should be considered as a limitation.

## Conclusions

Creating an immersive virtual reality environment that incorporates elements of Japanese garden aesthetics, Erickson's psychotherapy, relaxing music, and cognitive stimulation could potentially alleviate depressive symptoms in elderly women. The use of a similar device or application in hospital wards and nursing homes seems to be an affordable solution when there is a lack of constant psychological care and as a supplement to this care. Furthermore, outside the hospital ward, we can find the usefulness of similar solutions in reducing depressive symptoms, which appears to be particularly important in the context of supporting late-life depression therapy.

## Data Availability

The datasets generated during and/or analyzed during the current study are available from the corresponding author on reasonable request.
